# Cold-inducible RNA-binding protein through TLR4 signaling induces mitochondrial DNA fragmentation and regulates macrophage cell death after trauma

**DOI:** 10.1038/cddis.2017.187

**Published:** 2017-05-11

**Authors:** Zhigang Li, Erica K Fan, Jinghua Liu, Melanie J Scott, Yuehua Li, Song Li, Wen Xie, Timothy R Billiar, Mark A Wilson, Yong Jiang, Ping Wang, Jie Fan

**Affiliations:** 1 Department of Surgery, University of Pittsburgh School of Medicine, Pittsburgh, PA 15213, USA; 2 Research and Development, Veterans Affairs Pittsburgh Healthcare System, Pittsburgh, PA 15240, USA; 3 University of Pittsburgh School of Arts and Science, Pittsburgh, PA 15213, USA; 4 Guangdong Provincial Key Laboratory of Proteomics, State Key Laboratory of Organ Failure Research, Southern Medical University, Guangzhou 510515, China; 5 Department of Pharmaceutical Sciences, Center for Pharmacogenetics, University of Pittsburgh School of Pharmacy, Pittsburgh, PA 15261, USA; 6 McGowan Institute for Regenerative Medicine, University of Pittsburgh, Pittsburgh, PA 15219, USA; 7 The Feinstein Institute for Medical Research, Manhasset, NY 11030, USA

## Abstract

Trauma is a major cause of systemic inflammatory response syndrome and multiple organ dysfunction syndrome. Macrophages (M*ϕ*) direct trauma-induced inflammation, and M*ϕ* death critically influences the progression of the inflammatory response. In the current study, we explored an important role of trauma in inducing mitochondrial DNA (mtDNA) damage in M*ϕ* and the subsequent regulation of M*ϕ* death. Using an animal pseudo-fracture trauma model, we demonstrated that tissue damage induced NADPH oxidase activation and increased the release of reactive oxygen species via cold-inducible RNA-binding protein (CIRP)–TLR4–MyD88 signaling. This in turn, activates endonuclease G, which serves as an executor for the fragmentation of mtDNA in M*ϕ*. We further showed that fragmented mtDNA triggered both p62-related autophagy and necroptosis in M*ϕ*. However, autophagy activation also suppressed M*ϕ* necroptosis and pro-inflammatory responses. This study demonstrates a previously unidentified intracellular regulation of M*ϕ* homeostasis in response to trauma.

Traumatic injury represents a significant health care burden worldwide.^1^ Initial mortality is usually secondary to major neurologic injury or massive hemorrhage.^2, 3^ For those victims of severe traumatic injury who survive beyond the initial 24 h, major morbidities and mortality are often secondary to immune dysregulation, which includes increased inflammatory and innate immune signaling, as well as suppression of adaptive immune signaling. The net result leads to organ dysfunction and increased susceptibility to infection with the development of sepsis.^4, 5, 6, 7^ Unfortunately, we do not have effective medical intervention for these trauma patients, partly because of an incomplete understanding of the cellular determinants of immune dysregulation following trauma.

Macrophages (M*ϕ*), as a major innate immune cell population, direct trauma-induced inflammation, and M*ϕ* death critically influences the progression of inflammatory responses. M*ϕ* necroptosis has been reported as a response to infection, as well as sterile inflammatory stimuli such as LPS.^8, 9, 10, 11^ In turn, necroptotic M*ϕ* enhance inflammation through release of pro-inflammatory cell contents.^12^

Cold-inducible RNA-binding protein (CIRP) is a member of the cold shock protein family.^13^ Mouse and human CIRP contain 172 amino-acid residues (95% identical) that form a consensus sequence of N-terminal RAN-binding domain and C-terminal glycine-rich domain of nuclear proteins, which serve as RNA chaperones promoting RNA translation.^14, 15^ CIRP has been defined as an inflammatory mediator and a damage-associated molecular pattern (DAMP), which highly expresses during trauma and shock,^16, 17^ and induces a variety of cellular responses including the release of pro-inflammatory cytokines and endothelial dysfunction.^18^

In addition to supplying cellular energy, mitochondria are also involved with other functions including signal transduction, cell differentiation and death, and the maintenance of the cell cycle and cell growth control processes.^19^ In mammals, mitochondrial DNA (mtDNA) is circular with 15–17 000 base pairs encoding 37 genes, including 13 genes for respiratory complexes I, III, IV, and V.^20, 21^ Extracellular mtDNA is a known DAMP mediating inflammatory responses through a TLR9 signaling pathway during sepsis.^22, 23^ However, the role of intracellular-damaged mtDNA in the regulation of cellular homeostasis of innate immune cells has yet to be addressed.

Endonuclease G is an apoptotic DNase, located in the mitochondrial intermembrane space.^24^ The precursor of endonuclease G is an inactive 33 kDa protein, which must be activated by proteolytic cleavage to a 28 kDa form.^25^ Degradation of DNA by endonuclease G is independent of caspases.^26^ During animal development endonuclease G also functions to degrade mtDNA.^27^

In this study, we explored an important role for trauma in inducing mtDNA damage in M*ϕ*, but not in neutrophils, and subsequently regulating M*ϕ* death. Using an animal pseudofracture (PF) trauma model,^28^ we demonstrate that tissue damage acts through CIRP–TLR4–MyD88 signaling to induce NADPH oxidase activation, which increases release of reactive oxygen species (ROS) and activates endonuclease G. Endonuclease G serves as an executor to fragment mtDNA in the M*ϕ*, which we further demonstrate triggers both p62-related autophagy and necroptosis in M*ϕ*, although autophagy activation suppressed M*ϕ* necroptosis and pro-inflammatory responses. Our study therefore demonstrates a previously unidentified intracellular regulation pathway of M*ϕ* homeostasis in response to trauma.

## Results

### PF induces macrophage cytoplasmic DNA fragmentation

**Figure 1:**
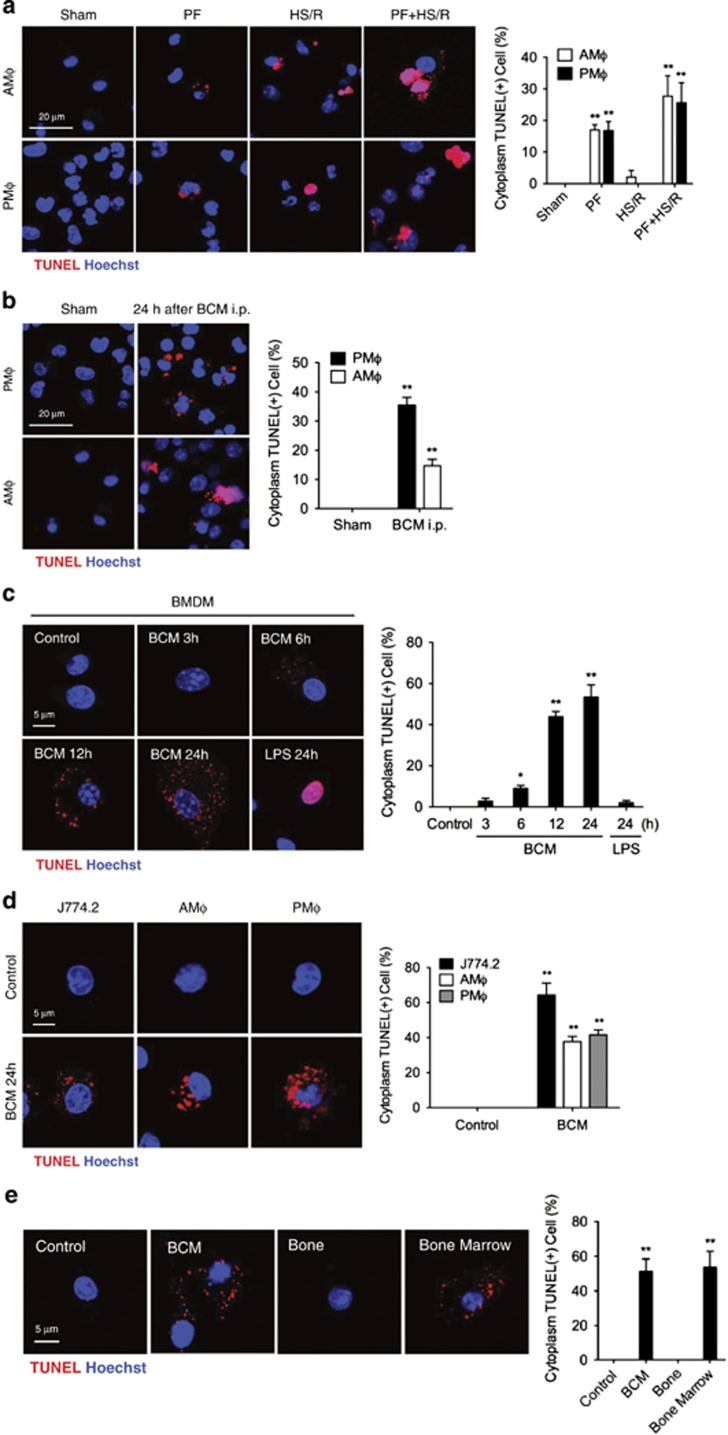
Pseudofracture induces macrophage cytoplasmic DNA fragmentation. (**a**) Alveolar macrophages (AM*ϕ*) and peritoneal macrophages (PM*ϕ*) from WT mice divided into one of the following: (1) sham for 24 h; (2) pseudofracture (PF) for 24 h; (3) hemorrhagic shock with resuscitation (HS/R) for 24 h; (4) PF with HS/R (PF+HS/R) for 24 h, and staining with TUNEL and Hoechst. Staining visualized by confocal microscopy and quantified. Cells were counted in three random fields for each independent experiment. (**b**) AM*ϕ* and PM*ϕ* from WT mice subjected to bone crush mixture (BCM; 6 ml/kg B.W. i.p.) for 24 h, and then stained with TUNEL and Hoechst, and visualized by confocal microscopy and quantified. (**c**) TUNEL and Hoechst staining in BMDM treated with 40 *μ*l/ml of BCM for 0–24 h, or treated with 1 *μ*g/ml LPS for 24 h. (**d**) TUNEL and Hoechst staining in J774.2 monocyte/M*ϕ* cell line, AM*ϕ* and PM*ϕ* treated with 40 *μ*l/ml of BCM for 24 h. Immunofluorescence imaged by confocal microscopy and quantified. (**e**) TUNEL and Hoeschst staining in BMDM treated with BCM, bone or bone marrow isolated from femur/tibia or WT mouse for 24 h. Confocal microscopy images also quantified. All results are representative of three independent experiments. The graphs show the mean and S.E.M., *n*=3. Significances between groups were determined by using independent samples two-tailed Student’s *t*-test. *P<0.05 or **P<0.01 compared with sham or control groups

To determine M*ϕ* DNA damage in response to trauma, WT mice were subjected to PF,^28^ a trauma model mimicking long bone fracture, or to hemorrhagic shock with resuscitation (HS/R), or to both PF and HS/R double hit. We used the TUNEL assay to detect DNA fragmentation as a marker of DNA damage. In the PF model, both alveolar macrophages (AM*ϕ*) and peritoneal macrophages (PM*ϕ*) exhibited TUNEL-positive dots in cytoplasm (Figure 1a). However, TUNEL staining colocalized with Hoechst stain (nuclear DNA) in HS/R model (Figure 1a). Furthermore, in mice subjected to the PF and HS/R double hit TUNEL staining localized to both the nucleus and cytoplasm (Figure 1a). These results suggest that HS/R and PF induce DNA fragmentation in M*ϕ* differently. To determine whether mediators in the bone crush mixture (BCM) used in the PF model contribute to the effect of PF on M*ϕ* cytoplasmic DNA fragmentation, BCM (6 ml/kg B.W.) was directly injected into the mouse peritoneal cavity. PM*ϕ* and AM*ϕ* were harvested at 24 h and DNA damage measured by TUNEL assay as above. As shown in Figure 1b, PM*ϕ*cytoplasmic DNA fragmentation significantly increased. Interestingly, BCM i.p. injection also*ϕ*-induced cytoplasmic DNA fragmentation in AM*ϕ*. This result suggests that BCM can induce cytoplasmic DNA fragmentation in both proximal and distal M*ϖ*.

The *in vivo* study was also recapitulated *in vitro*. Bone marrow-derived macrophage (BMDM) were treated with BCM (40 *μ*l/ml) for 24 h, and as shown in Figure 1c, the percentage of M*ϕ* with TUNEL-positive cytoplasm increased significantly by 6 h after BCM treatment, and peaked at 24 h. However, LPS-induced DNA fragmentation was localized to the nucleus, which was different from BCM-induced cytoplasmic DNA fragmentation (Figure 1c). We also directly treated different M*ϖ* with BCM *in vitro*. As shown in Figure 1d, BCM-induced cytoplasmic DNA fragmentation in J774.2 mouse monocyte cell line, and AM*ϖ*, and PM*ϕ*. We further separated the BCM into two parts, bone and bone marrow, and found that only the bone marrow was able to induce M*ϕ* cytoplasmic DNA fragmentation (Figure 1e). Collectively, these results suggest that PF induces M*ϕ* cytoplasmic DNA fragmentation and it is bone marrow components of the BCM that are responsible for these effects.

### Damaged tissue induces macrophage mtDNA fragmentation

**Figure 2:**
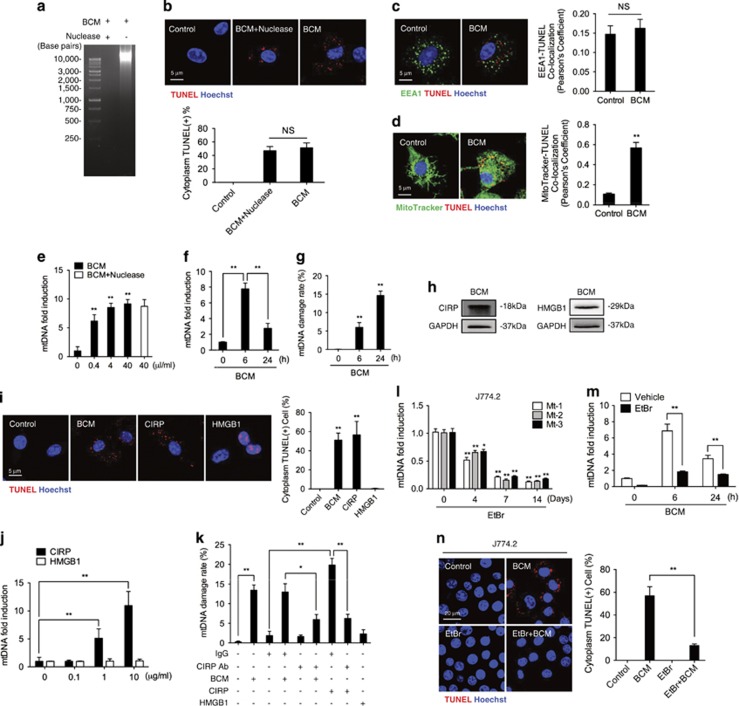
Damaged tissue induces macrophage mtDNA fragmentation. (**a**) Separation of DNA fragments by agarose gel electrophoresis in BCM treated with or without nuclease. (**b**) Confocal images and quantification of TUNEL and Hoechst staining of BMDM treated for 24 h with nuclease pretreated or non-pretreated BCM (40 *μ*l/ml). (**c**) Immunofluorescence images showing the colocalization and quantification of colocalization of endosome (EEA1; green) and fragmented DNA (TUNEL; red) in BMDM treated with 40 *μ*l/ml BCM for 24 h. (**d**) Confocal immunofluorescence images showing colocalization and quantification of colocalization of mitochondria (MitoTracker; green) and fragmented DNA (TUNEL; red) in BMDM treated with BCM (40 *μ*l/ml) for 24 h. (**e**) Quantification of fold induction of mtDNA measured by RT-PCR in BMDM treated with 0–40 *μ*l/ml BCM or 40 *μ*l/ml nuclease-pretreated BCM for 6 h. (**f**) mtDNA fold induction and (**g**) mtDNA damage measured by RT-PCR in BMDM treated with BCM (40 *μ*l/ml) for 0, 6, or 24 h. (**h**) Western blot for CIRP and HMGB1 expression in BCM. (**i**) Confocal images and quantification of TUNEL and Hoechst staining of BMDM challenged with BCM (40 *μ*l/ml), CIRP (10 *μ*g/ml), or HMGB1 (10 *μ*g/ml) for 24 h. (**j**) mtDNA fold induction measured by RT-PCR in BMDM treated with 0–10 *μ*g/ml CIRP or HMGB1 for 6 h. (**k**) mtDNA damage measured by RT-PCR in BMDM stimulated with BCM (40 *μ*l/ml), CIRP (10 *μ*g/ml), or HMGB1 (10 *μ*g/ml), which combined with or without IgG isotype antibody (10 *μ*g/ml) or CIRP neutralizing antibody (10 *μ*g/ml) for 24 h. (**l**) Quantification of mtDNA by RT-PCR with three specific mitochondrial primers (Mt-1/2/3) in J774.2 cells treated with 100 ng/ml ethidium bromide (EtBr) for 0–14 days. (**m**) mtDNA fold induction quantified by RT-PCR in J774.2 cells +/− pretreatment with 100 ng/ml EtBr for 7 days, and stimulation with BCM (40 *μ*l/ml) for 0, 6, and 24 h. (**n**) Confocal images and quantification of fragemented DNA (TUNEL; red) and Hoechst nuclear staining of J774.2 cells +/− pretreatment with 100 ng/ml EtBr for 7 days, and stimulated with BCM (40 *μ*l/ml) for 0, 6, and 24 h. All results are representative of three independent experiments. The graph shows the mean and S.E.M., *n*=3. Significances between groups were determined by using independent samples two-tailed Student’s *t*-test. *P<0.05 or **P<0.01 *versus* the control or indicates groups. NS, no significant difference

In order to determine the source of fragmented DNA and exclude exogenous DNA, BCM was pretreated with nuclease to remove DNA/RNA. As shown in Figure 2a, nuclease successfully cleared DNA from BCM. However, nuclease-pretreated BCM was still able to induce BMDM cytoplasmic TUNEL dots similarly to non-pretreated groups (Figure 2b). M*ϕ* endocytosis of exogenous DNA is commonly localized to endosome,^29^ so we therefore detected colocalization of fragmented DNA with endosomes that were visualized by staining early endosome antigen 1 (EEA1), which localizes early endosomes and is required for fusion of early and late endosomes. As Figure 2c shows, the fragmented DNA does not localize to endosomes, suggesting an endogenous source of fragmented DNA. Next, we determined whether endogenous fragmented DNA derived from mitochondria. Confocal microscopy showed that BCM-induced fragmented DNA partly colocalized with mitochondria, as stained by Mitotracker, and partly localized to areas surrounding mitochondria suggesting mitochondrial origin (Figure 2d). Both BCM and nuclease-pretreated BCM also increased levels of mtDNA by 6 h (Figure 2e), but this increase was reversed by 24 h (Figure 2f). To calculate the mtDNA damage rate, as shown in Figure 2g, BCM induces about 5% of total mtDNA damage at 6 h and about 15% mtDNA damage at 24 h.

BCM is essentially a damaged tissue containing multiple DAMPs including the major DAMPs CIRP and HMGB1 as detected with immunoblots (Figure 2h). To address which of these two DAMPs is the major component inducing macrophage mtDNA fragmentation, we stimulated BMDM with recombinant CIRP or HMGB1. Recombinant CIRP induced TUNEL-positive BMDM cytoplasm similarly to BCM, but recombinant HMGB1 did not have the same effects (Figure 2i). CIRP (10 *μ*g/ml), rather than recombinant HMGB1, significantly induced increased levels of mtDNA by 6 h. (Figure 2j). Furthermore, while CIRP-induced mtDNA damage increased, CIRP neutralizing antibody reduced the BCM-induced mtDNA damage as compared to the group treated with nonspecific IgG isotype antibody (Figure 2k). The recombinant HMGB1, again, did not induce the mtDNA damage (Figure 2k).

To further define the role of the mtDNA responses to BCM, we generated mtDNA-depleted M*ϕ*. Previous studies showed that treatment of J774.2 M*ϕ* with ethidium bromide (EtBr) for 7–14 days effectively depletes mtDNA.^30, 31, 32^ We confirmed mtDNA levels in the J774.2 cells decreased to ~10% after 100 ng/ml of EtBr treatment for 7–14 days using three different pairs of mtDNA primers (see Materials and Methods), which produced consistent results showing mtDNA depletion (Figure 2l). In depleted cells, BCM failed to induce the large increase in mtDNA seen in Figure 2m. When visualized, levels of fragmented mtDNA at 24 h after BCM treatment decreased in EtBr pretreated J774.2 cells (Figure 2n). These results indicate that damaged tissue induces M*ϕ* mtDNA fragmentation, and CIRP, but not HMGB1, serves as a DAMP molecule inducing these changes.

### BCM-induced mtDNA fragmentation is mediated through TLR4–MyD88 signaling

**Figure 3:**
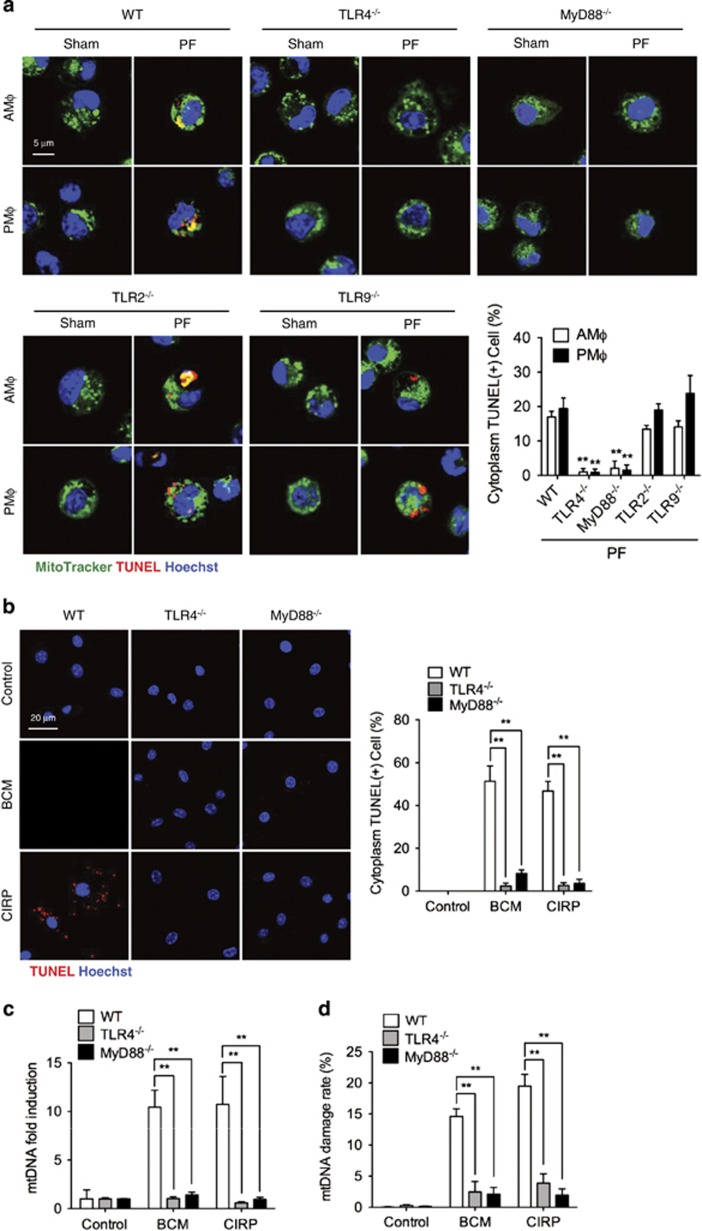
BCM-induced mtDNA fragmentation is mediated by TLR4–MyD88 signaling. (**a**) Confocal images and analysis of AM*ϕ* and PM*ϕ* harvested from WT, TLR4^−/−^, MyD88^−/−^, TLR2^−/−^, and TLR9^−/−^ mice 24 h after PF, and stained with MitoTracker (mitochondria; green), TUNEL (fragmented DNA; red), and Hoechst nuclear stain (blue). (**b**) Confocal images and analysis of TUNEL (fragmented DNA; red) and Hoechst nuclear stain (blue) in BMDM isolated from WT, TLR4^−/−^, or MyD88^−/−^ mice and treated with BCM (40 *μ*l/ml) or CIRP (10 *μ*g/ml) for 24 h. (**c**) mtDNA fold induction and (**d**) mtDNA damage measured by RT-PCR in WT, TLR4^−/−^, or MyD88^−/−^ BMDM treated with BCM (40 *μ*l/ml) for 0, 6, and 24 h. All results are representative of three independent experiments. The graphs show the mean and S.E.M., *n*=3. Significances between groups were determined by using independent samples two-tailed Student’s *t*-test. *P<0.05 or **P<0.01 compares with WT groups or between the indicated groups

CIRP has been reported to signal via TLR4–MyD88 signaling pathways to promote inflammation.^17, 33^ To address whether PF, through TLR4–MyD88 signaling, induces mtDNA fragmentation, we used TLR4^−/−^, MyD88^−/−^, TLR2^−/−^, and TLR9^−/−^ mice. As shown in Figure 3a, PF-induced mtDNA fragmentation is diminished in M*ϕ* from TLR4^−/−^ and MyD88^−/−^ mice, but not in M*ϕ* from TLR2^−/−^ and TLR9^−/−^ mice. Similar results were also observed *in vitro*, with significantly decreased cytoplasmic TUNEL-positive TLR4^−/−^ or MyD88^−/−^ BMDM treated with BCM or CIRP (Figure 3b). Consistently, BCM- or CIRP-induced mtDNA levels at 6 h (Figure 3c) and mtDNA damage at 24 h (Figure 3d) were significantly diminished in TLR4^−/−^ and MyD88^−/−^ BMDM. These results suggest that BCM and its component CIRP act through the TLR4–MyD88 signaling pathway to induce mtDNA fragmentation.

### ROS mediates mtDNA fragmentation

**Figure 4:**
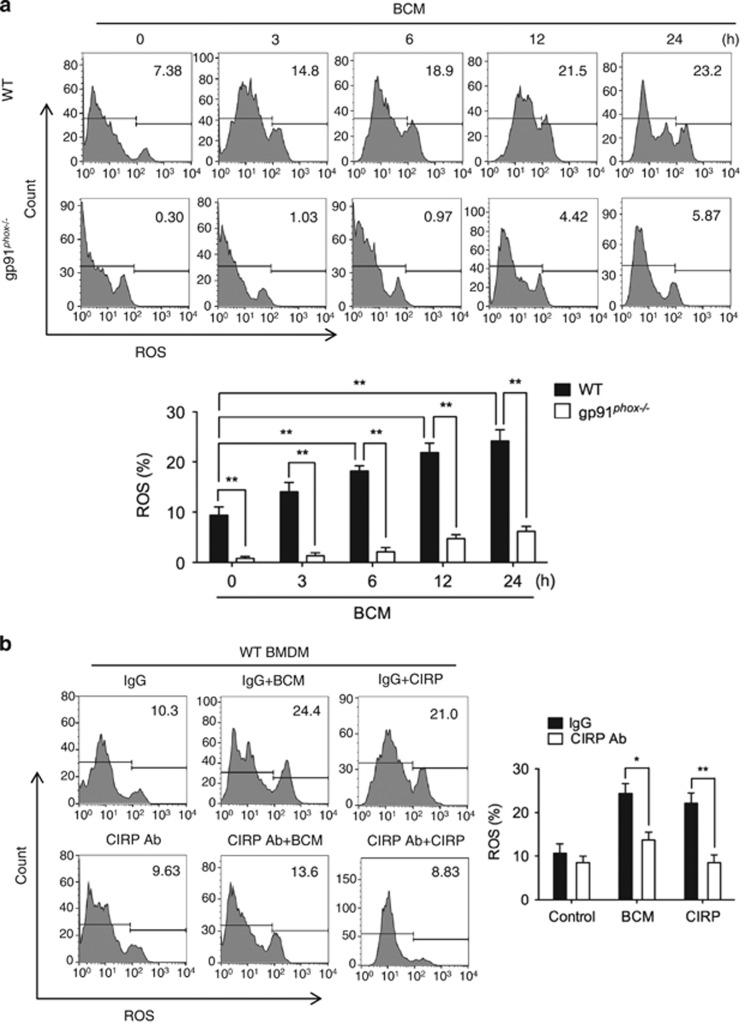

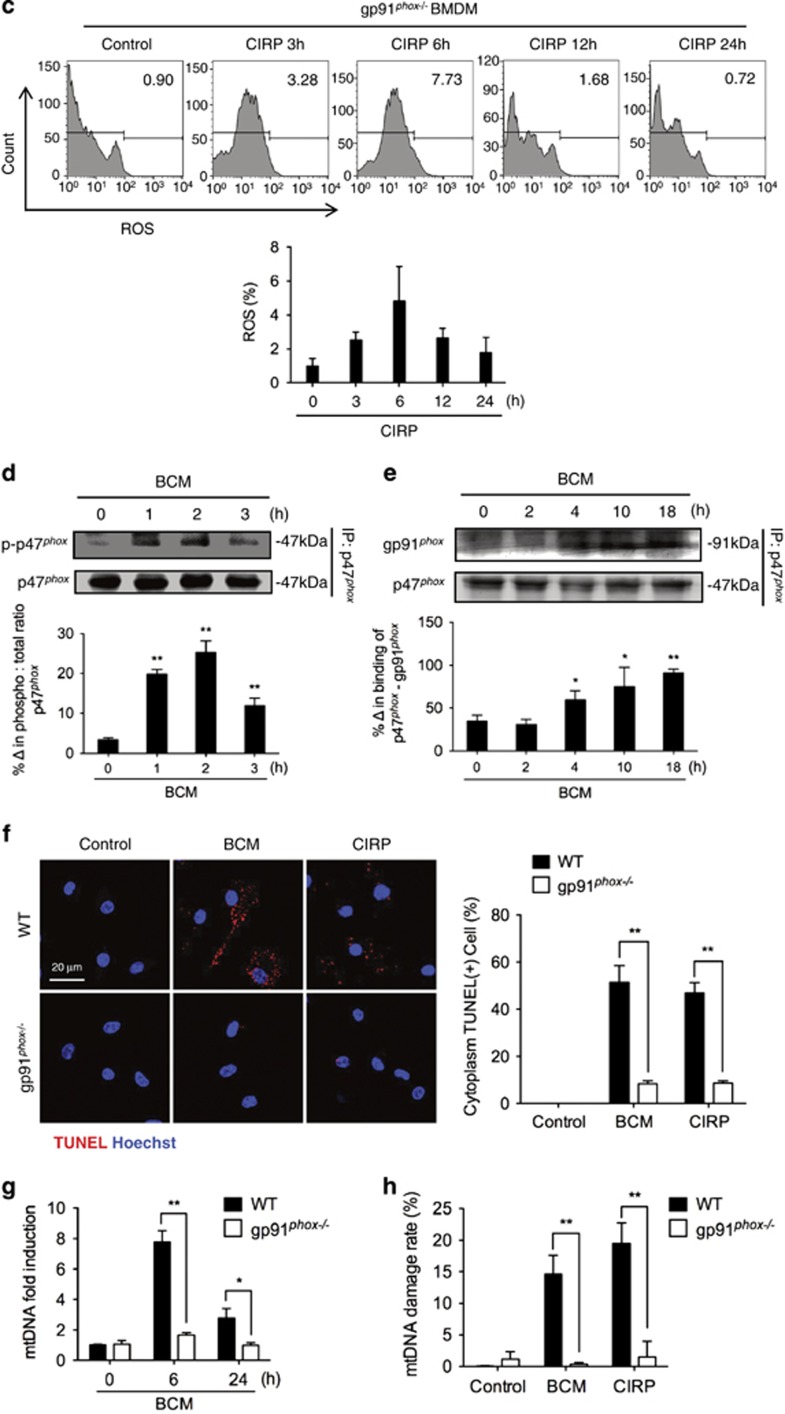
ROS mediates mtDNA fragmentation. (**a**) Flow cytometric plots and analysis of DCF fluorescence (ROS) in WT or gp91^*phox*−/−^ BMDM stimulated with 40 *μ*l/ml of BCM for 0–24 h. (**b**) Flow cytometric plots and analysis of DCF fluorescence in WT BMDM treated with 40 *μ*l/ml BCM or 10 *μ*g/ml CIRP for 24 h +/− IgG isotype antibody (10 *μ*g/ml) or CIRP neutralizing antibody (10 *μ*g/ml). (**c**) Flow cytometric plots and analysis of DCF fluorescence (ROS) in gp91^*phox*−/−^ BMDM treated with 10 *μ*g/ml CIRP for 0–24 h. (**d**) Western blot to show p47^*phox*^ phosphorylation up to 3 h after stimulation with BCM (40 *μ*l/ml). (**e**) Immunoprecipitation (IP) with anti-p47^*phox*^ and immunoblot for gp91^*phox*^ in lysates from WT BMDM treated with BCM for 0–18 h. (**f**) Confocal images and analysis of TUNEL and Hoechst staining in WT or gp91^*phox*−/−^ BMDM stimulated by 40 *μ*l/ml BCM or 10 *μ*g/ml CIRP for 24 h. (**g**) mtDNA fold induction and (**h**) mtDNA damage measured by RT-PCR in WT or gp91^*phox*−/−^ BMDM treated with BCM (40 *μ*l/ml) for 0, 6, and 24 h. All results are representative of three independent experiments. The graphs show the mean and S.E.M., *n*=3. Significances between groups were determined by using independent samples two-tailed Student’s *t*-test. *P<0.05 or **P<0.01 compared with control groups or indicated groups

Previous studies showed that oxidative stress induces mitochondrial dysfunction and mtDNA damages.^34, 35^ The NADPH oxidases from NOX family act as a key ROS producers in various cells.^36, 37^ To address whether NADPH oxidase-derived ROS mediate BCM-induced mtDNA damage, we subjected WT or gp91^*phox*−/−^ BMDM to challenge with BCM for 0–24 h. Gp91^*phox*^ is one of the five subunits of NADPH oxidase, and gp91^*phox*−/−^ cells do not have NADPH oxidase activity.^38^ As shown in Figure 4a, BCM induced WT BMDM ROS increase after 6 h, as measured by CM-H2DCFDA, a general oxidative stress indicator, while the gp91^*phox*−/−^ prevented BCM-induced intracellular ROS increase. This suggests that NADPH oxidase is one of the important ROS sources induced by BCM. In addition, CIRP neutralizing antibody significantly decreased the BCM-induced ROS production in WT BMDM (Figure 4b). Furthermore, recombinant CIRP induced ROS increase in WT BMDM, while this was significantly decreased in gp91^*phox*−/−^ BMDM given CIRP for up to 24 h (Figure 4c). We also measured NADPH oxidase activation in BMDM, by measuring p47^*phox*^ phosphorylation and p47^*phox*^ association with gp91^*phox*^ by immunoprecipitation (IP). As shown in Figures 4d and e, BCM induced p47^*phox*^ phosphorylation in WT BMDM at as early as 1 h, and the association of p47^*phox*^ with gp91^*phox*^ increased by 4 h after BCM treatment. Furthermore, gp91^*phox*−/−^ BMDM had significantly reduced levels of TUNEL-positive cytoplasm after stimulation with either BCM or CIRP (Figure 4f). BMDM from gp91^*phox*−/−^ mice had reduced levels of mtDNA compared with WT BMDM (Figure 4g) and reduced levels of mtDNA damage after BCM or CIRP treatment (Figure 4h). These results clearly indicate that NADPH oxidase-derived ROS play an important role in the mechanism underlying BCM-induced mtDNA damage.

### Endonuclease G fragments mtDNA in M*ϕ* after BCM treatment

**Figure 5:**
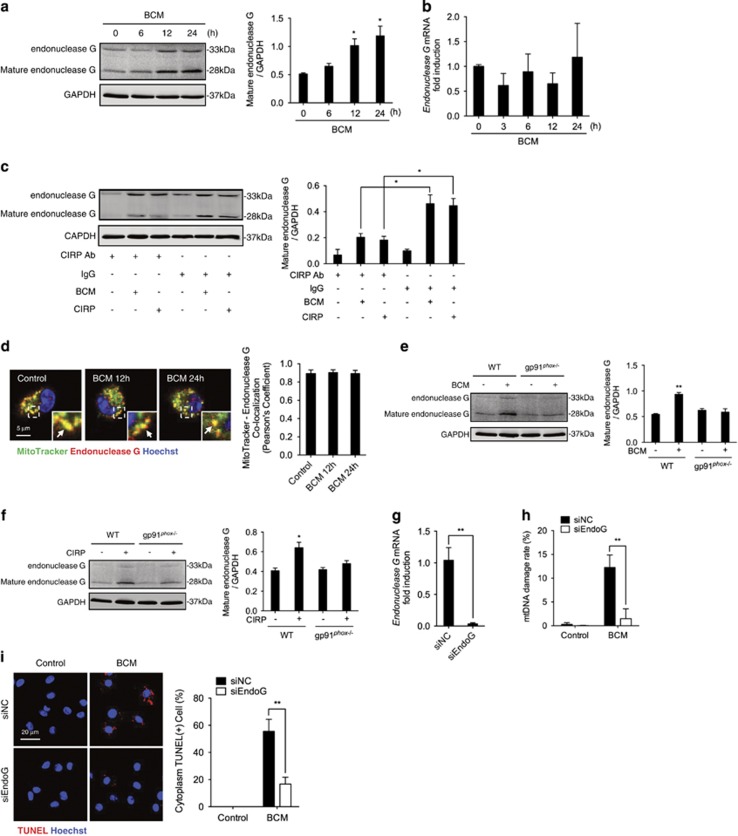
Endonuclease G fragments mtDNA in M*ϕ* after BCM treatment. (**a**) Western blot of whole-cell lysates showing endonuclease G protein expression and (**b**) mRNA expression in BMDM stimulated by 40 *μ*l/ml BCM for 0–24 h. (**c**) Western blot of whole-cell lysates showing endonuclease G protein expression in BMDM treated with 40 *μ*l/ml BCM or 10 *μ*g/ml CIRP for 24 h +/− CIRP neutralizing antibody (10 *μ*g/ml) or IgG isotype antibody (10 *μ*g/ml). (**d**) Confocal immunofluorescence images and analysis of colocalization of mitochondria (MitoTracker; green) and endonuclease G (red) in BMDM treated with 40 *μ*l/ml BCM for 12 or 24 h. Arrows indicate the colocalization of mitochondria and endonuclease G. (**e**) Western blot of whole-cell lysates showing endonuclease G expression in WT or gp91^*phox*−/−^ BMDM stimulated by 40 *μ*l/ml BCM or (**f**) stimulated with 10 *μ*g/ml CIRP for 24 h. (**g**) Endonucleases G mRNA expression in BMDM transfected with control non-coding siRNA (siNC) or siRNA specific for endonuclease G (siEndoG) for 24 h. (**h**) mtDNA damage and (**i**) confocal images and analysis of TUNEL staining (fragmented DNA; red) and nuclear Hoechst staining (blue) in WT BMDM transfected with siNC or siEndoG and stimulated by 40 *μ*l/ml BCM for 24 h. All results are representative of three independent experiments. The graphs show the mean and S.E.M., *n*=3. Significances between groups were determined by using independent samples two-tailed Student’s *t*-test. *P<0.05 or **P<0.01 compared with control groups or indicated groups

Previous studies reported that endonuclease G, which locates at mitochondrial inner membrane, fragments chromosomal DNA in caspase-independent apoptosis.^26^ Furthermore, the endonuclease G also degrades mtDNA during animal development.^27, 39^ To determine the role of endonuclease G in mtDNA fragmentation in M*ϕ*, we measured changes in endonuclease G protein and mRNA levels after BCM stimulation. As shown in Figure 5a, BCM increased protein expression of mature endonuclease G (28 kDa) by 12 h after BCM stimulation. Interestingly, endonuclease G mRNA level was not significantly changed (Figure 5b). CIRP recombinant protein increased mature endonuclease G expression, while CIRP neutralizing antibody prevented the BCM- and CIRP-induced increase (Figure 5c). We also determined the cellular location of endonuclease G in WT BMDM after BCM stimulation (Figure 5d) and show that it colocalizes with mitochondria both before and after BCM stimulation.

To determine whether the endonuclease G expression is dependent on NADPH oxidase, we compared protein levels of mature endonuclease G in WT and gp91^*phox*−/−^ BMDM in response to BCM or CIRP. BMDM from gp91^*phox*−/−^ mice challenged with BCM (Figure 5e) or CIRP (Figure 5f) exhibited markedly lower level of mature endonuclease G expression compared to WT BMDM. Furthermore, we investigated the role of endonuclease G in mtDNA fragmentation using endonuclease G knockdown approach. Transfection of small interfering RNA against endonuclease G (siEndoG) successfully knocked down endonuclease G in BMDM as shown by very low levels of endonuclease G mRNA (Figure 5g). Transfection with siEndoG significantly decreased BCM-induced mtDNA damage (Figure 5h) and TUNEL-positive cytoplasmic staining (Figure 5i) as compared with BMDM transfected with control non-coding siRNA (siNC). These data indicate that NADPH oxidase induces increased expression of mature endonuclease G, which mediates BCM-induced mtDNA damage, therefore suggesting that endonuclease G is the executor of mtDNA fragmentation.

### Fragmented mtDNA induces M*ϕ* autophagy

**Figure 6:**
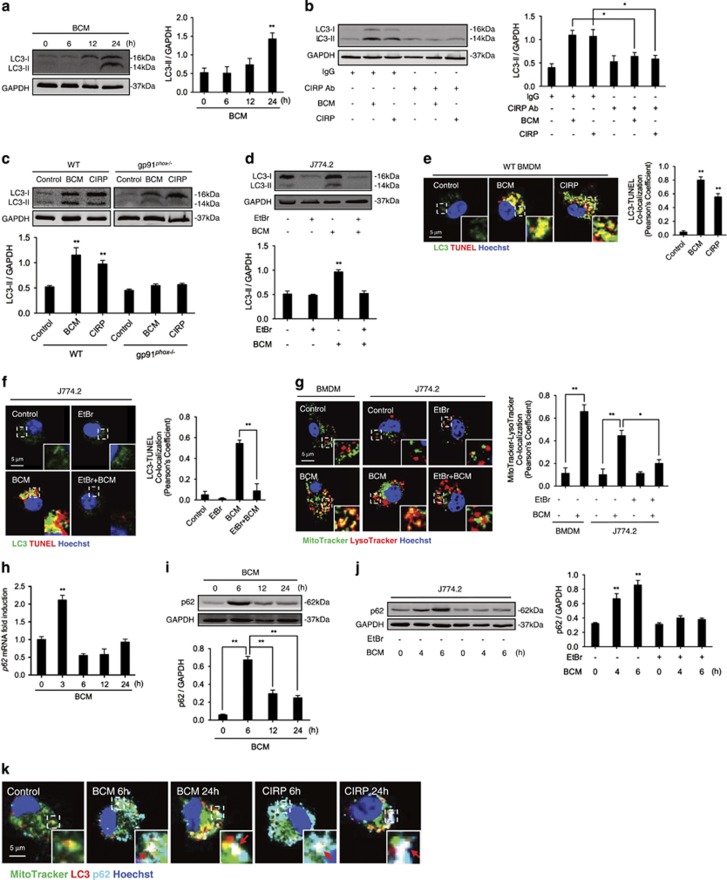
Fragmented mtDNA induces macrophage autophagy. (**a**) Western blot of whole-cell lysates showing LC3 expression in BMDM stimulated by 40 *μ*l/ml BCM for 0–24 h. GAPDH used as loading control. (**b**) Western blot of whole-cell lysates showing LC3 expression in BMDM treated with 40 *μ*l/ml BCM or 10 *μ*g/ml CIRP for 24 h +/− IgG isotype antibody (10 *μ*g/ml) or CIRP neutralizing antibody (10 *μ*g/ml). (**c**) Western blot of whole-cell lysates showing LC3 expession in WT or gp91^*phox*−/−^ BMDM stimulated by 40 *μ*l/ml BCM or 10 *μ*g/ml CIRP for 24 h. (**d**) Western blot of whole-cell lysates showing LC3 expression in J774.2 cells +/− pretreatment with 100 ng/ml EtBr for 7 days, and stimulated with BCM (40 *μ*l/ml) for 24 h. (**e**) Confocal immunofluorescence images and analysis showing colocalization of LC3 (autophagosome; green) and TUNEL (fragmented DNA; red) and Hoechst nuclear stain (blue) in BMDM treated with 40 *μ*l/ml BCM or 10 *μ*g/ml CIRP for 24 h. (**f**) Confocal immunofluorescence images and analysis of colocalization of LC3 (autophagosome; green) and TUNEL (fragmented DNA; red) and Hoechst nuclear stain (blue) in J774.2 cells +/− pretreatment with 100 ng/ml EtBr for 7 days, and stimulated with BCM (40 *μ*l/ml) for 24 h. (**g**) Confocal immunofluorescence images showing colocalization of mitochondria (MitoTracker; green) and lysosomes (LysoTracker; red) in BMDM, or J774.2 cells +/− pretreatment with 100 ng/ml EtBr for 7 days, and stimulated with BCM (40 *μ*l/ml) for 24 h. (**h**) Expression levels of p62 mRNA or (**i**) protein expression in BMDM stimulated by BCM (40 *μ*l/ml) for 0–24 h. (**j**) Western blot of whole-cell lysates showing p62 protein expression in J774.2 cells +/− pretreatment with 100 ng/ml EtBr for 7 days, then stimulated with BCM (40 *μ*l/ml) for 0–6 h. (**k**) Confocal immunofluorescence images and analysis of colocalization of mitochondria (MitoTracker; green), LC3(autophagosomes; red) and p62 (cyan) in BMDM stimulated with BCM (40 *μ*l/ml) or CIRP (10 *μ*g/ml) for 24 h. Arrows indicate the colocalization of mitochondria-LC3-p62 (white). All results are representative of three independent experiments. The graphs show the mean and S.E.M., *n*=3. Significances between groups were determined by using independent samples two-tailed Student’s *t*-test. *P<0.05 and **P<0.01 *versus* the control or compared between the indicated groups

Autophagy refers to a cell self-regulation process, allowing orderly degradation and recycling of abnormal cell components.^40^ Autophagy is known as a major regulator of removal of damaged mitochondria through mitophagy.^41, 42^ Next, we addressed whether BCM-induced mtDNA fragmentation activates autophagy. First, a time-course study showed that BCM treatment of BMDM increased LC3-II expression at 24 h (Figure 6a). LC3-II is the activated form of LC3 that is incorporated in autophagosomes.^43^ Second, CIRP neutralizing antibody suppressed LC3-II formation in response to BCM and CIRP (Figure 6b). Third, there was very little formation of LC3-II in gp91^*phox*−/−^ BMDM after BCM or CIRP treatment compared with WT BMDM (Figure 6c). Similarly, mtDNA depletion in J774.2 cells prevented BCM-induced LC3-II formation (Figure 6d). To confirm our findings, we also visualized colocalization of LC3 with TUNEL staining in the BMDM treated with BCM or CIRP. Figure 6e shows that both BCM and CIRP can induce LC3 colocalization with TUNEL-positive fragmented DNA. As expected from our results above, when mtDNA was depleted in J774.2 cells, there was no TUNEL staining in the cytoplasm, and so no localization of TUNEL with LC3 (Figure 6f). These observations suggest fragmented mtDNA induces and localizes to the autophagosome.

In addition, as Figure 6g shows, BCM also induced mitophagy in BMDM, which can be identified as colocalization of mitochondria (MitoTracker) and lysosomes (LysoTracker). However, this induction of mitophagy was prevented in mtDNA-depleted J774.2 cells (Figure 6g).

The degradation of p62 is often used as an indicator of autophagy.^44^ BCM induced increases in expression of p62 mRNA at 3 h (Figure 6h) and in p62 protein expression at 6 h (Figure 6i), which is then followed by decreases in p62 protein expression at 12 and 24 h suggesting activation of autophagy pathways and clearance of p62 (Figure 6i). However, in mtDNA-depleted J774.2 cells BCM failed to induce p62 upregulation (Figure 6j). Again, colocalization of mitochondria, LC3, and p62 following BCM or CIRP stimulation for 24 h was visualized by confocal microscopy as shown in Figure 6k. Taken together, these data suggest that BCM- and CIRP-induced mtDNA fragmentation leads to mitophagy.

### Autophagy prevents fragmented mtDNA-induced macrophage necroptosis

**Figure 7:**
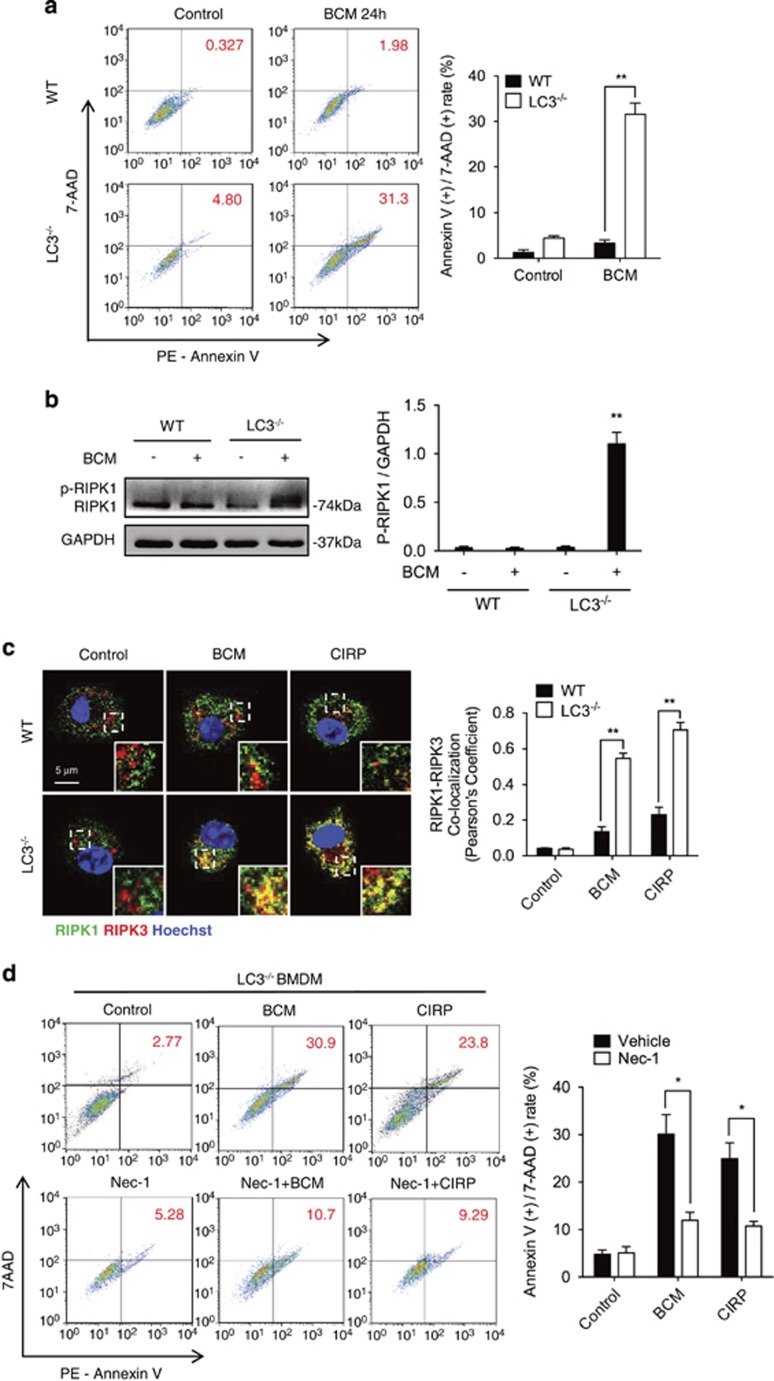
Autophagy prevents fragmented mtDNA-induced macrophage necroptosis. (**a**) Flow cytometric plots and analysis of cell death (AnnexinV-positive, 7-AAD-positive) in WT or LC3^−/−^ BMDM stimulated by 40 *μ*l/ml BCM for 24 h. (**b**) Western blot of whole-cell lysates showing RIPK1 phosphorylation (p-RIPK1) in WT or LC3^−/−^ BMDM stimulated by 40 *μ*l/ml BCM for 24 h. (**c**) Confocal microscopy images and analysis showing colocalization of RIPK1 (green) and RIPK3 (red) in WT or LC3^−/−^ BMDM treated with BCM (40 *μ*l/ml) or CIRP (10 *μ*g/ml) for 24 h. (**d**) Flow cytometric plots and analysis of cell death (AnnexinV-positive, 7-AAD-positive) in LC3^−/−^ BMDM treated with BCM (40 *μ*l/ml) or CIRP (10 *μ*g/ml) +/− 30 *μ*M necrostatin-1 (Nec-1) for 24 h. All results are representative of three independent experiments. The graphs show the mean and S.E.M., *n*=3. Significances between groups were determined by using independent samples two-tailed Student’s *t*-test. *P<0.05 or **P<0.01 compared with control groups or indicated groups

Autophagy is one of the critical mechanisms for the clearance of damaged mtDNA, so it is important for cellular homeostasis.^45^ We next determined the role of autophagy in regulation of cell fate in response to mtDNA fragmentation. In LC3^−/−^ BMDM, in which autophagosomes cannot be induced by mtDNA fragmentation, BCM-induced cell death significantly increased compared with WT BMDM (Figure 7a). To further define the type of the cell death occurring, we measured RIPK1 phosphorylation, as well as colocalization of RIPK1 and RIPK3, which are features of necroptosis.^46^ We found that BCM treatment induced higher levels of RIPK1 phosphorylation in LC3^−/−^ BMDM (Figure 7b), and increased colocalization of RIPK1 and RIPK3 compared with WT BMDM (Figure 7c). Addition of the necroptosis inhibitor necrostatin-1 (Nec-1) significantly blocked BCM- or CIRP-induced cell death in LC3^−/−^ BMDM (Figure 7d). These data reveal an important role for autophagy in preventing necroptosis following mtDNA damage.

### Necroptotic M*ϕ* induce inflammatory responses in naive M*ϕ*

**Figure 8:**
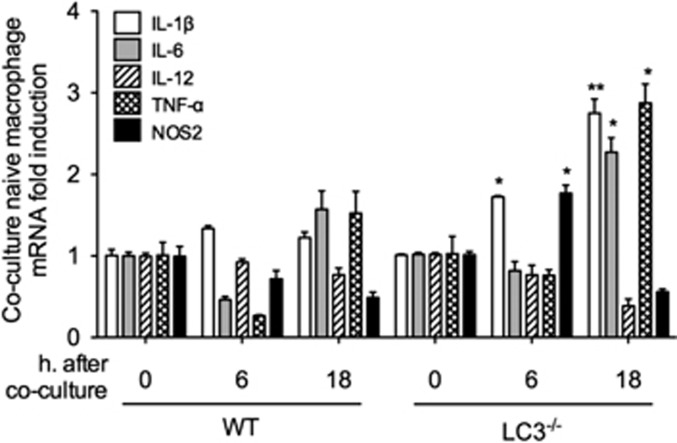
Necroptotic M*ϕ* induce inflammatory responses in naive M*ϕ*. mRNA expression of IL1*β*, IL-6, IL-12, TNF-*α*, and NOS2 in naive BMDM placed in the bottom of a Transwell plate, with WT or LC3^−/−^ BMDM pre-stimulated by BCM (40 *μ*l/ml) for 24 h in the upper wells. Cells were then cocultured for 0–18 h. All results are representative of three independent experiments. The graphs show the mean and S.E.M., *n*=3. Significances between groups were determined by using independent samples two-tailed Student’s *t*-test. *P<0.05 and **P<0.01 compared with control groups

BCM induced necroptosis in LC3^−/−^ BMDM and necroptosis is well known as a form of inflammatory cell death.^47, 48^ To determine the significance of mtDNA fragmentation-induced autophagy in maintaining cellular homeostasis, we investigated the effect of necroptotic M*ϕ* on neighboring healthy M*ϕ*. WT or LC3^−/−^ BMDM were pretreated with BCM for 24 h, and then were cocultured with naive macrophages for 6 or 18 h. As shown in Figure 8, the BCM-pretreated LC3^−/−^ BMDM, which lack autophagy and exhibit necroptosis, significantly increased inflammatory responses in naive macrophages, including increased IL-1*β* and NOS2 mRNA expression at 6 h, and increased IL-1*β*, IL-6 and TNF-*α* mRNA at 18 h. These results suggest that mtDNA fragmentation-induced autophagy plays a protective role in limiting local inflammation through the regulation of cell death.

## Discussion

**Figure 9:**
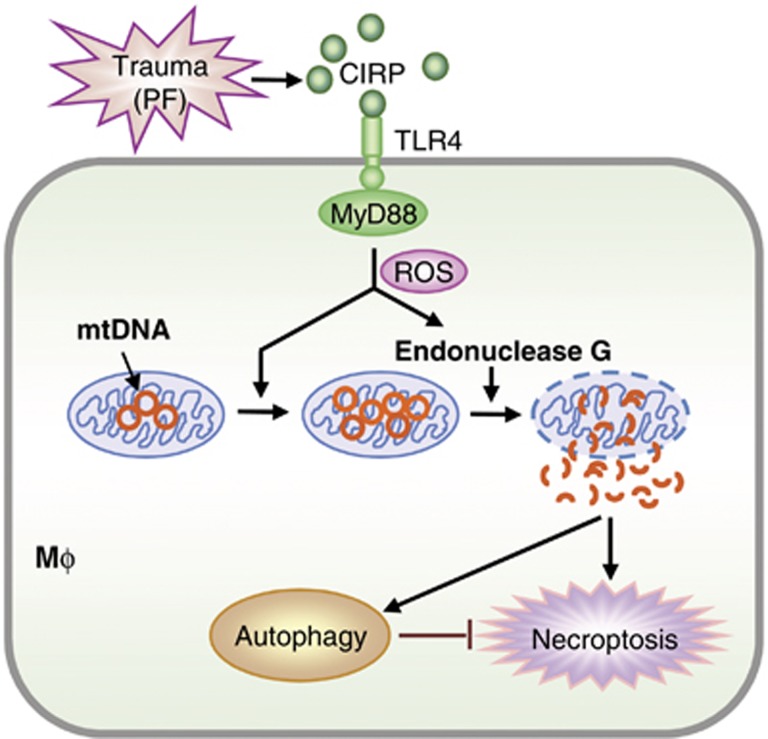
Model of trauma-induced mtDNA fragmentation regulating macrophage death. PF or BCM through CIRP–TLR4–MyD88 signaling induces NADPH oxidase activation and release of ROS, which activate endonuclease G. Endonuclease G directly fragments mtDNA, which triggers M*ϕ* autophagy, as well as necroptosis by separate pathways. However, autophagy also suppresses M*ϕ* necroptosis to limit local inflammation

The mechanisms behind trauma/tissue damage regulation of innate immune responses are not yet fully defined.^16^ Our current study explored a mechanism underlying trauma-induced regulation of M*ϕ* death, in which CIRP released from damaged tissue acts through TLR4–MyD88 signaling to induce M*ϕ* mtDNA fragmentation, via a pathway in which NADPH oxidase-derived ROS served as a major mediator for the induction of endonuclease G, which, in turn, directly mediates mtDNA fragmentation. Fragmented mtDNA then triggered M*ϕ* autophagy and necroptosis through separate pathways, although autophagy also suppressed M*ϕ* necroptosis, to attenuate propagation of local inflammation (Figure 9).

Previous studies have shown that during injury extracellular mtDNA serves as a DAMP that induces inflammatory response through TLR9 signaling.^23, 49, 50^ However, the mechanism of intracellular mtDNA damage had not been fully identified. In our current study, we revealed an important role for intracellular fragmented mtDNA in regulating M*ϕ* death and inflammation.

We defined fragmented DNA in M*ϕ* cytoplasm after BCM treatment as damaged mtDNA based on the facts that: (1) predeleting exogenous DNA in BCM using nuclease did not prevent the formation of fragmented DNA in M*ϕ* cytoplasm (Figures 2a and b); (2) fragmented DNA was not endosomal, suggesting it had not been endocytosed (Figure 2c); (3) DNA-free recombinant CIRP induced cytoplasmic fragmented DNA in M*ϕ* (Figures 2i and j); (4) BCM treatment induced changes in mtDNA synthesis and degradation (Figures 2e–g); and (5) depletion of mtDNA with EtBr prevented cytoplasmic fragmented DNA in M*ϕ* in response to BCM (Figures 2l–n). We also demonstrated a dynamic alteration in mtDNA quantity after BCM treatment with increased mtDNA in the early phase (6 h), followed by decreased levels in the late phase (24 h). Overall we detected about 15% mtDNA damage, an indication of mtDNA fragmentation. The observations that CIRP induced mtDNA fragmentation and neutralizing antibody against CIRP prevented the mtDNA fragmentation in response to BCM indicate that CIRP is one of the major components in damaged tissue to induce mtDNA fragmentation.

Our previous studies showed that TLR4–MyD88 signaling mediates NADPH oxidase activation in neutrophils and lung vascular endothelial cells in response to hemorrhagic shock and LPS.^37, 51, 52^ In the current study, we showed that the PF/tissue damage activation of TLR4–MyD88 signaling results in increases in ROS production through the activation of NADPH oxidase in M*ϕ*. It is noticeable that LPS, CIRP, and HMGB1, all being TLR4 agonists, induce very different responses in terms of DNA damage. For example, LPS causes DNA fragmentation in the nucleus, and CIRP in the mitochondria; HMGB1 did induce DNA fragmentation in the nucleus, though perhaps not as much as LPS. This raises a fundamental biological question on how limited receptor classes can induce such a broad range of different cellular responses, which has generated several hypotheses. A plausible hypothesis is that following receptor activation, different signaling pathways selectively adopt different adaptor proteins to induce different downstream outcomes. It can be assumed that CIRP, HMGB1, and LPS bind to different domain structures of TLR4, which then induce different TLR4 conformational changes and activate different signaling pathways. Indeed, this is an open question that remains to be further elucidated.

The identity of the executor protein downstream of ROS that cleaved mtDNA was not clear.^35^ Previous studies showed that endonuclease G can be activated under oxidative stress^53, 54, 55^ and then functions as a chromosomal DNA fragmentation enzyme.^26, 56^ Studies have also shown that endonuclease G plays a critical role in paternal mtDNA degradation during animal development.^27, 39^ In this study, we demonstrated that the endonuclease G is an important executor for induction of mtDNA fragmentation, and ROS is required for its activation. Genetic deletion of the NADPH oxidase subunit gp91^*phox*^ significantly diminished BCM- and CIRP-induced endonuclease G activation, and knockdown of endonuclease G prevented BCM-induced mtDNA damage and appearance of mtDNA fragmentation in M*ϕ* cytoplasm.

The current study further determined the protective effect of autophagy in PF/tissue damage. We identified that fragmented mtDNA induces autophagy, which serves as a mechanism to clear the damaged mtDNA. In LC3^−/−^ BMDM, which cannot initiate the autophagosome formation, the damaged mtDNA promotes the M*ϕ* necroptosis, which propagates and enhances inflammatory responses by activating neighboring innate immune cells. These observations indicate an important role for autophagy in cellular homeostasis during trauma/tissue damage through the limitation of local inflammation.

In summary, this study demonstrates a novel mechanism underlying trauma-induced mtDNA damage and subsequent self-regulation of cell death and inflammation aiming to maintain cellular homeostasis. Targeting the signaling revealed in this study might serve as a potential approach of therapeutic intervention for the treatment of post-trauma inflammation.

## Materials and methods

### Animal strains

C57BL/6 wild-type (WT) mice, gp91^*phox*^ knockout (gp91^*phox*−/−^) mice, and LC3 knockout (LC3^−/−^) mice were purchased from The Jackson Laboratory (Bar Harbor, ME, USA). TLR2 knockout (TLR2^−/−^) mice, TLR4 knockout (TLR4^−/−^) mice, TLR9 knockout (TLR9^−/−^) mice, and myeloid differentiation primary response gene 88 knockout (MyD88^−/−^) mice were obtained from Dr Billiar’s laboratory at the University of Pittsburgh. All animal experimental protocols were reviewed and approved by the Institutional Animal Care and Use Committees of University of Pittsburgh and VA Pittsburgh Healthcare System.

### Reagents

Primary antibodies for cell staining were: EEA1 (endosomal marker) antibody (2411S, Cell Signaling Technology, Danvers, MA, USA), LC3 antibody (4599S, Cell Signaling Technology), RIPK1 antibody (610458, BD Biosciences, San Jose, CA, USA), RIPK3 antibody (sc-135170, Santa Cruz Biotechnology, Dallas, TX, USA), MitoTracker and LysoTracker (M7514 and L7528, Thermo Fisher Scientific, Pittsburgh, PA, USA), p62/SQSTM1 Antibody (MAB8028, R&D Systems, Minneapolis, MN, USA). Secondary antibodies including Alexa Fluor 488-conjugated anti-mouse IgG, Cy5-conjugated anti-mouse IgG, Alexa Fluor 488-conjugated anti-rabbit IgG, and Cy3-conjugated anti-rabbit IgG were provided by the Center for Biologic Imaging, University of Pittsburgh Medicine Center.

Primary antibodies used for western blotting include CIRP antibody (from Dr Ping Wang, the Feinstein Institute for Medical Research),^17^ HMGB1 antibody (ab18256, Abcam, Cambridge, MA, USA), anti-RIPK1 antibody (610458, BD Biosciences), anti-phosphoserine antibody (61–8100, Thermo Fisher Scientific), p47^*phox*^ antibody (sc-14015, Santa Cruz Biotechnology), gp91^*phox*^ antibody (sc-5827, Santa Cruz Biotechnology), Endonuclease G antibody (#4969, Cell Signaling Technology), LC3 antibody (#4599, Cell Signaling Technology), p62/SQSTM1 antibody (MAB8028, R&D Systems), GAPDH (D16H11) XP Rabbit mAb (#5174 Cell Signaling Technology).

*In Situ* Cell Death Detection Kit, TMR red (TUNEL) was from Roche (12156792910, Indianapolis, IN, USA). Annexin V detection kit was from BD Biosciences. MitoTracker Green FM was from Thermo Fisher Scientific; Dynabeads Protein G Immunoprecipitation Kit (10007D) for immunoprecipitation was from Thermo Fisher Scientific. Transfect reagents, Lipofectamine LTX Reagent with PLUS Reagent (15338100) was purchased from Thermo Fisher Scientific; siNC and siEndoG was purchased from Integrated DNA Technologies (Coralville, IA, USA); Necroptosis inhibitor Necrostatin-1 (BML-AP309-0020) was purchased from Enzo Life Sciences (Farmingdale, NY, USA). Recombinant HMGB1 (1690-HMB-050) was from R&D Systems. Rabbit IgG isotype control (ab171870) was purchased from Abcam. Recombinant mouse CIRP protein and anti-CIRP antibody were from Dr Ping Wang, the Feinstein Institute for Medical Research.^17^ The recombinant mouse CIRP protein was detected a residual ~10 pg of LPS per *μ*g of CIRP by the Limulus amebocyte lysate assay.^17^ And the anti-CIRP Ab was raised in New Zealand white rabbits by standard procedures at Covance (Princeton, NJ, USA).^17^

### Mouse PF model

The mouse PF model was carried out as previously described.^28, 57^ Briefly, femurs and tibias were collected from a killed donor mice and then crushed using a sterile mortar and pestle. An amount of 0.5 g of the bone crush material was homogenized in 2 ml sterile phosphate-buffered solution (PBS) to prepare the BCM. The recipient mouse was anesthetized with ketamine (50 mg/kg B.W.) combined with xylazine (5 mg/kg B.W.) and the thighs were squeezed with a hemostat for 30 s to induce a soft tissue lesion, followed by injection of 0.15 ml of the BCM in the tissue lesion area. Sham animals underwent the same anesthesia procedure and injection of 0.15 ml of normal saline at the thighs.

### BMDM isolation and culture

Bone marrow was flushed with prechilled Dulbecco’s Modified Eagle Medium (DMEM) from femurs and tibias, which were harvested from WT or gene knockout mice following the previously described method.^58^ Briefly, cell pellets were collected by centrifugation at 4 °C, and erythrocytes were lysed with RBC lysis buffer (eBioscience, San Diego, CA, USA). The resultant cells were then washed two times with PBS and suspended in BMDM culture medium (DMEM containing 10% FBS complemented with 50 *μ*g/ml penicillin/streptomycin and 10 ng/ml recombinant macrophage-colony stimulating factor (M-CSF; Sigma-Aldrich, St Louis, MO, USA)) at a concentration of 10^6^ cells per ml and seeded into 6-cm ultra-low attachment surface plates (Corning Costar, Corning, NY, USA). The BMDM culture medium was changed on day 3 and day 5. BMDM were fully differentiated and ready for use at day 7.

### Cell staining

BMDM were seeded in a glass-bottomed Petri dish (P35G-0-10-C, MatTek Corporation, Ashland, MA, USA) and fixed with 4% paraformaldehyde for 15 min at room temperature. After washing with PBS, cells were permeabilized with 0.01% Triton X-100 in PBS for 15 min at room temperature, followed by blocking with 5% bovine serum albumin in PBS for 1 h at room temperature. The cells were stained with TUNEL following manufacturer’s instructions or were incubated with a primary antibody at 4 °C overnight, followed by incubation with fluorescence-conjugated secondary antibody for 1 h at room temperature. Cell nucleus was stained with Hoechst 33258 (Sigma-Aldrich). The BMDM were then measured by confocal microscopy (Olympus, Fluoview-FV1000, Olympus America Co., Center Valley, PA, USA). Cells were counted in three random fields in each independent experiment. We then quantified the colocalization of the target proteins by Pearson’s coefficient using ImageJ version 1.50i.

### Western blot

BMDM lysates were separated by 8 and 15% SDS-PAGE, and then transferred onto PVDF membranes. After blocking for 1 h at room temperature with blocking buffer (LI-COR Biosciences, Lincoln, NE, USA), blots were incubated with primary antibody at 4 °C overnight, followed by incubation with appropriate secondary antibodies (LI-COR Biosciences) for 1 h. Protein bands were detected using the Odyssey System from LI-COR Biosciences and the intensity of each band was quantified using ImageJ version 1.50i. The intensity of target protein band was normalized with reference protein band and calculated for the fold changing.

### DNA extraction, mtDNA quantitative and damage rate calculation

Total DNA was isolated from BMDM using DNAzol DNA Isolation Reagents (Thermo Fisher Scientific) by following the instructions. Real-time PCR was done using the iTaq Universal SYBR Green Supermix (1725121, Bio-Rad, Hercules, CA, USA) in a Bio-Rad iQ5 real-time PCR machine (Bio-Rad). The following gene-specific primers were used for amplifying genes: *Mt-1* forward, 5′- GCCGTACTGCTCCTATTATCACTA -3′, and reverse, 5′-AGAATCAGAACAGATGCTGGTAGA-3′ *Mt-2* forward, 5′-TGATGGTACGGACGAACAGA-3′, and reverse, 5′-GCCTTCTATTGCTGATGGTAGTC-3′ *Mt-3* forward, 5′-GCCCCAGATATAGCATTCCC-3′, and reverse, 5′-GTTCATCCTGTTCCTGCTCC-3′ and *GAPDH* forward, 5′-AACCTGCCAAGTATGATGA-3′ and reverse, 5′-GGAGTTGCTGTTGAAGTC-3′. The forward and reverse sequences of *Mt-1*, *Mt-2*, and *Mt-3* have been checked on NCBI BLAST for bacterial homology. All of these sequences do not match the gene sequences of microbes. The BLAST results show that *Mt-1* and *Mt-3* targets mouse mtDNA cytochrome oxidase subunit 1 gene sequences, and *Mt-2* targets mouse mtDNA NADH dehydrogenase subunit 5 gene sequences.

Amplification was performed with cycling conditions of 95 °C for 15 s then 60 °C for 30 s for 40 cycles. After the amplification protocol was completed, PCR product was subjected to melt–curve analysis using Bio-Rad iQ5 software (Bio-Rad). Fold change was calculated using the ΔΔ threshold cycle method and the value for the GAPDH gene, which was normalized to untreated groups. mtDNA damage was calculated by the ratio of long and short segments amplified from mtDNA PCR production. The primers used were: long segments primers- *mt long* forward, 5′-GCCAGCCTGACCCATAGCCATAATAT-3′, and reverse, 5′-GAGAGATTTTATGGGTGTAATGCGG-3′ short segments primers-*mt short* forward, 5′-CCCAGCTACTACCATCATTCAAGT-3′, and reverse, 5′-GATGGTTTGGGAGATTGGTTGATGT-3′ Reference segments primers: *β-Globin* forward, 5′-TTGAGACTGTGATTGGCAATGCCT-3′, and reverse, 5′-CCTTTAATGCCCATCCCGGACT-3′.^59^ PCR was done using the TaKaRa Ex Taq DNA polymerase (Clontech Laboratories, Inc., Mountain View, CA, USA) in Applied Biosystems PCR machine (Thermo Fisher Scientific). Amplification was performed with cycling conditions of 94 °C for 30 s then 55 °C for 60 s and 72 °C for 10 min for 30 cycles. PCR product was quantified by using of NanoDrop spectrophotometer (NanoDrop, Wilmington, DE, USA). The mtDNA long and short PCR production normalized to *β*-Globin, respectively, and calculated the mtDNA damage rate (%) using the following formula:


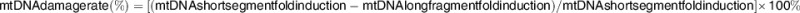


### RNA extraction and quantitative real-time PCR

The cells form co-culture bottom wells BMDM were harvested and total RNA was isolated by using TRIzol RNA Isolation Reagents (Thermo Fisher Scientific) by following the instructions. Real-time RT-PCR was done using iTaq Universal SYBR Green Supermix (1725121, Bio-Rad) in a Bio-Rad iQ5 real-time PCR machine (Bio-Rad). The following gene-specific primers were used for amplifying genes: *p62* forward, 5′-AGGATGGGGACTTGGTTGC-3′, and reverse, 5′-TCACAGATCACATTGGGGTGC-3′ *Endonuclease G* forward, 5′-GGAAGTCCTATGTGAAGTA-3′, and reverse, 5′-ATCAGCACCTTGAAGAAG-3′ *IL-1β* forward, 5′-GAAATGCCACCTTTTGACATG-3′, and reverse, 5′-TGGATGCTCTCATCAGGACAG-3′ *IL-6* forward, 5′-CCAAGAGGTGAGTGAGTGCTTCCC-3′, and reverse, 5′-CTGTTGTTCAGACTCTCTCCCT-3′ *IL-12* forward, 5′-AAGTGGAAGACATTAAGGAAGAA-3′, and reverse, 5′-CCAACCAAGCAGAAGACA-3′ *TNF-α* forward, 5′-GACGTGGAACTGGCAGAAGAG-3′, and reverse, 5′-TTGGTGGTTTGTGAGTGTGAG-3′ *NOS2* forward, 5′-CCGCCGCTCTAATACTTA-3′, and reverse, 5′-TTCATCAAGGAATTATACAGGAA-3′ and *18S* forward, 5′-GTAACCCGTTGAACCCCATT-3′ and reverse, 5′-CCATCCAATCGGTAGTAGCG-3′. Reverse transcription was done using iScript Reverse Transcription Supermix (170–8840, Bio-Rad) following the manufacturer’s instructions. Amplification was performed with cycling conditions of 95 °C for 15 s then 60 °C for 30 s for 40 cycles. After the amplification protocol was completed, PCR product was subjected to melt–curve analysis using Bio-Rad iQ5 software (Bio-Rad). Fold change was calculated using the ΔΔ threshold cycle method^60^ and the value for the 18S gene, which was normalized to untreated groups.

### Immunoprecipitation and detection of phosphorylated p47^*phox*^ and p47^*phox*^ - gp91^*phox*^ association

BMDM were lysed in cell lysis buffer (9803, Cell Signaling Technology, Danvers, MA, USA) with protease inhibitor cocktails and PMSF (P8340 and 93482, Sigma-Aldrich). The lysed cell protein was immunoprecipitated with anti-p47^*phox*^ antibody (sc-14015, Santa Cruz Biotechnology) using Dynabeads protein G immunoprecipitation kit (10007D, Thermo Fisher Scientific). The immunoprecipitated proteins were then subjected to immunoblotting analysis using anti-phosphoserine antibody (61–8100, Thermo Fisher Scientific) and anti-gp91^*phox*^ antibody (sc-5827, Santa Cruz Biotechnology), respectively.

### Data presentation and statistical analysis

The data are presented as mean±S.E.M. of the indicated number of experiments. SPSS 19.0 was used for statistical analysis. Significances between groups were determined by using independent samples two-tailed Student’s *t*-test. *P*<0.05 was considered as statistically significant.
